# A systematic literature review of cyber-security data repositories and performance assessment metrics for semi-supervised learning

**DOI:** 10.1007/s44248-023-00003-x

**Published:** 2023-04-06

**Authors:** Paul K. Mvula, Paula Branco, Guy-Vincent Jourdan, Herna L. Viktor

**Affiliations:** Present Address: School of Electrical Engineering and Computer Science (EECS), University of Ottawa, 800 King Edward Avenue, Ottawa, K1N 6N5 ON Canada

**Keywords:** Cyber-security, Datasets, Performance metrics, Phishing detection, Intrusion detection, Malware detection

## Abstract

In Machine Learning, the datasets used to build models are one of the main factors limiting what these models can achieve and how good their predictive performance is. Machine Learning applications for cyber-security or computer security are numerous including cyber threat mitigation and security infrastructure enhancement through pattern recognition, real-time attack detection, and in-depth penetration testing. Therefore, for these applications in particular, the datasets used to build the models must be carefully thought to be representative of real-world data. However, because of the scarcity of labelled data and the cost of manually labelling positive examples, there is a growing corpus of literature utilizing Semi-Supervised Learning with cyber-security data repositories. In this work, we provide a comprehensive overview of publicly available data repositories and datasets used for building computer security or cyber-security systems based on Semi-Supervised Learning, where only a few labels are necessary or available for building strong models. We highlight the strengths and limitations of the data repositories and sets and provide an analysis of the performance assessment metrics used to evaluate the built models. Finally, we discuss open challenges and provide future research directions for using cyber-security datasets and evaluating models built upon them.

## Introduction

As a result of the significant technological advancements made throughout the years, people’s lifestyles are shifting from traditional to more electronic. This shift has resulted in an increase in cybercrimes on the Internet. Therefore, adequate measures have to be put in place to secure computer systems. Moreover, computer security or cyber-security systems must be capable of detecting and preventing cyber-attacks in real-time. The intersection of the Machine Learning (ML) and cyber-security fields has recently been rapidly growing as researchers make use of either fully labelled datasets with Supervised Learning (SL), unlabeled datasets with Unsupervised Learning (UL) or combining labelled and unlabeled data with Semi-Supervised Learning (SSL) to identify the various types of cyber-attacks. Due to the high cost and scarcity of labelled data in the cyber-security domain, SSL applications for cyber-security tasks have gained traction. Several datasets have been made available to the public to build ML-based defensive mechanisms. In ML, the quality of the output is determined by the quality of the input [1]; in other words, for ML models to generalize effectively, the datasets upon which they are built must be representative of real-world data. Therefore, surveys on the available datasets and performance evaluation metrics used to build and evaluate SSL models are required to give up-to-date information on recent cyber-security datasets and suitable performance metrics used in SSL frameworks to provide a starting point for new researchers who wish to investigate this vital subject.

Several works focusing on cyber-security provide discussions of datasets and data repositories that can be used for building ML models. For instance, Ring et al. [2] presented an extensive survey on network-based intrusion detection datasets discussing datasets containing packet-based, flow-based and neither packet- nor flow-based data while Glass-Vanderlan et al. [3] focused on Host-Based Intrusion Detection Systems (HIDS) and touched upon datasets and sources mainly related to HIDS. Other articles described datasets for (i) intrusion, malware and spam detection (e.g. [4–8]); (ii) network anomaly detection (e.g. [9]); or (iii) phishing URL detection (e.g. [10]). However, these works often focus on a particular cyber-security domain and do not examine in detail the characteristics of the available datasets and the performance evaluation metrics that are suitable for the various research challenges.

Because of the expanding interest in this area and the rapid speed of research, these surveys quickly become outdated; there is, therefore, an obvious need for a comprehensive survey to present the most recent datasets and evaluation metrics and their usage in the literature. To fill this gap, we present an exhaustive evaluation of the cyber-security datasets used to build SSL models. In this paper, we conduct a systematic literature review (SLR) of publicly available cyber-security datasets and performance assessment metrics used for building and evaluating SSL models. To this end, we provide a summary of datasets used to construct models for cyber-security-related tasks; the covered areas include not only network- and host-based intrusion detection, but also spam and phishing detection, Sybil and botnet detection, Internet traffic and domain name classification, malware detection and categorization, and power grid attacks detection. Additionally, we examine the performance assessment metrics used to evaluate the SSL models and discuss their usage in the selected papers. Furthermore, we provide a list of datasets, tools, and resources used to collect and analyze the data that have been made publicly available in the literature. Finally, we provide a discussion on the open research challenges and a list of observations with regard to datasets and performance metrics. This is, to the best of our knowledge, the first SLR analyzing a wide array of cyber-security datasets and performance evaluation metrics for SSL tasks, as well as providing easy access to publicly available datasets.

Our key contributions are the following:

1. We provide a description of the most commonly used SSL techniques.
2. We provide insights on the major cybercrimes for which SSL solutions have been explored.
3. We present a systematic literature review of the publicly available cyber-security datasets, repositories and performance evaluation metrics used.
4. We analyze the open challenges found in the literature and provide a set of recommendations for future research.

The remaining sections are organized as follows. Section 2 presents the definitions, important concepts, and basic assumptions of SSL, as well as a brief introduction to the methods utilized in the literature we reviewed and an overview of the different cybercrimes the included articles’ authors propose to counter. Additionally, we provide examples that highlight successful industrial deployments of ML for countering cyber threats, demonstrating the practical applications of the methods discussed in the literature. In Sect. 3, we present the methodology we used to construct our survey and in Sect. 4, an in-depth analysis of the publicly available datasets and the different evaluation metrics used in the selected papers is presented. Section 5 discusses the open challenges faced by the reviewed methods applying SSL for cyber-security, with respect to the datasets and evaluation metrics, presents a set of observations and the lessons learned, and highlights strategies for bridging the gap between research and practice. Finally, Sect. 6 concludes the work.

## Background on SSL and cyber-security

Machine Learning (ML), the core subset of Artificial Intelligence (AI), may be defined as the systematic study of computer algorithms and systems that allow computer programs to automatically improve their knowledge or performance through experience [11]. It is a branch of computer science where the goal is to teach computers with sample data, i.e., training data, to make predictions or decisions on unseen data. ML algorithms can be categorized into three main types: SL, UL, and Reinforcement Learning (RL). In SL, the task, i.e., the inference of the function to map input data points from an instance space to their corresponding labels in the output space using labelled examples [12, 13], can either be classification where the function being learned is discrete, i.e., input data points in the input space are mapped to categorical values, or regression where the function being learned is continuous, i.e., input data points are mapped to real values. In contrast to SL, in UL, there are no labels available, therefore the goal of UL algorithms is to capture important patterns or extract relationships from untagged (unlabeled) data as probability density distributions [14] and in RL, the algorithms’ goal is to attempt to maximize the feedback (reward) they are provided with. SSL conceptually stands between SL and UL, [15–17]. Out-of-core Learning (OL), or Incremental, or Online Learning, is a learning technique where the data becomes available in a sequential, one at a time, manner [18]. In OL, the model can learn from newly available data, in addition to making predictions from it. Information Technology (IT) security, Computer security or simply cyber-security is the protection of computer systems and networks from cyber-attacks, i.e., information disclosure, loss, theft, or damage to their hardware, software, or electronic data, as well as from the disruption or misdirection of the services they offer [19].

SSL and ML, in general, have brought significant benefits to the cyber-security domain, including improved detection capabilities, adaptive learning, automation, and threat intelligence [20] (see Sect. 2.3 for industrial examples). However, there are also challenges that need to be addressed, including the lack of quality data, adversarial attacks, model explainability, and bias and discrimination [21, 22]. Addressing these challenges will be critical to ensuring that ML remains a useful tool in the fight against cyber threats.

In the remainder of this section, we introduce the key principles and techniques of SSL, provide a summary of cybercrimes examined in the literature, and present examples that demonstrate the potential of ML in mitigating cyber threats in the real world.

### SSL concepts and methods

We will first introduce some notations. Let $\mathcal {D}_L=(x_i,l(x_i ))_{i=1}^k$ denote a labelled dataset where each sample $(x_i,l(x_i))$ consists of data point $x_i$ from the instance space $\mathcal {X}$ and a target variable $l(x_i)$ in the output space $\mathcal {Y}$. Let $\mathcal {D}_U=(x_i)_{i=k+1}^{k+u}$ denote an unlabeled dataset. In SL, when $l(x_i)$ consists of categorical values we face a classification task and when it consists of real values we have a regression task. In UL, the model is only provided with unlabeled data, i.e., $\mathcal {D}_U$. SL can build strong models to predict labels for unlabeled samples, but it requires $\mathcal {D}_L$ to contain diverse samples manually labelled by domain experts, which may not only be too costly but may also contain inaccurate labels due to human mistakes. Therefore, in practice, $u \gg k$. On the other hand, even though UL does not require labelled samples to infer patterns, it is prone to overfitting. SSL makes use of both $\mathcal {D}_L$ and $\mathcal {D}_U$ to infer a function whose performance surpasses one built with either SL or UL by making use of at least one of the main learning assumptions, i.e., smoothness, low-density, manifold, [23], and cluster, [24], assumption.

The smoothness assumption is based on the notion that if two data points, $x_1$ and $x_2$, lie close in the instance space, $\mathcal {X}$, their corresponding class labels, $l(x_1)$ and $l(x_2)$, should also be close (the same), in the output space $\mathcal {Y}$; the transitivity assumption, that states that if $x_1$ lies close to $x_2$ and $x_2$ lies close to $x_3$, then $x_1$ lies transitively close to $x_3$, is an important idea in the smoothness assumption because “close points in $\mathcal {X}$ have the same label,” thus this assumption implies that if $x_2$ is a noisy version of $x_1$, they should still have the same predicted label. In the low-density assumption, it is implied that data points with the same label are clustered in high-density sections of the instance space, i.e., the decision boundary must pass through a low-density region, $\mathcal {R} \subset \mathcal {X}$, and the probability of any data point, $p(x_i)$, being in the low-density region is low, i.e., $p(x_i)$ in $\mathcal {R}$ is low. This also verifies that the smoothness assumption is satisfied. In the manifold assumption, the instance space, $\mathcal {X}$, consists of one or more Riemannian manifolds $\mathcal {M}$ on which samples share the same label. According to the cluster assumption, which can be seen as a generalization of the other three assumptions mentioned earlier [16], if data points are in the same cluster, they are likely to share the same label, and there may be several clusters constituting the same class [15].

**Fig. 1:**
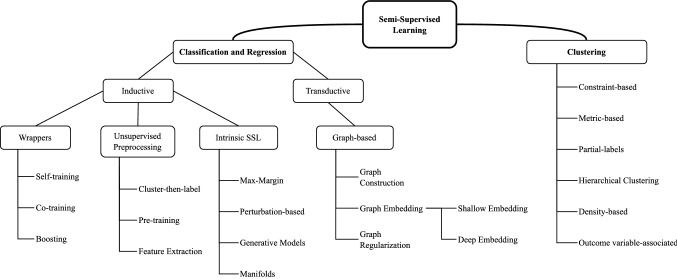
Taxonomy of SSL techniques (adapted from [16, 25, 26])

Based on [16, 25, 26], the taxonomy in Fig. 1 provides a general overview of the SSL approaches which will be described in more detail in Sects. 2.1.1 and 2.1.2. An overview of the key concepts in the taxonomy is presented next.

SS Classification and Regression methods can either be transductive or inductive [15, 27, 28]. In inductive SSL, the model is first built using information from $\mathcal {D}_L$ and $\mathcal {D}_U$ and it can then be used as one built with SL to generate predictions for previously unseen, unlabeled samples; there exists a clear distinction between a training phase and a testing phase. In transductive SSL, on the other hand, the goal is to generate labels for the unlabeled samples fed to the learner, therefore there is no clear distinction between a training and testing phase. Frequently, transductive approaches create a graph across all data points, including labelled and unlabeled, expressing the pairwise similarity of data points with weighted edges and are incapable of handling additional unseen data [17]. We group both SS Classification and Regression because they predict output values for input samples but note that most SS Classification approaches are incompatible with SS Regression, and we, therefore, specify when they may be compatible in Sect. 2.1.1.

In the SS Clustering assumption, the learner’s goal is clustering but a small amount of knowledge is available in the form of constraints, must-link constraints (two samples must be within the same cluster) and cannot-link constraints (two data points cannot be within the same cluster). It differs from traditional clustering in the way the constraints are accommodated: either by biasing the search for relevant clusters or altering a distance or similarity metric [29]. When it is not possible for an SL method to work, even in a transductive form, because the available knowledge is too far from being representative of a target classification of the items, the cluster assumption may allow the use of the available knowledge to guide the clustering process [30]. Bair [25] provides a survey on SS Clustering methods and groups them into constraint-based, partial-labels, SS hierarchical clustering and outcome variable associated methods.

A plethora of SSL approaches have been proposed in the literature, each making use of at least one of the SSL assumptions described. The following sections briefly describe the frequently used SSL methods showing how they relate to the SSL assumptions.

#### SSL for classification and regression

We divide the classification and regression methods between the two main classes: inductive SSL and transductive SSL.

##### Inductive methods

The goal of inductive methods is to build a model from labelled and unlabeled data and use the model as a built-in SL (only with labelled data) to make predictions on unlabeled data. Inductive methods can further be divided into wrapper methods, unsupervised preprocessing, and intrinsically semi-supervised methods. In wrapper methods, one or more supervised-based learners are first trained based on the labelled data only, then the learner or set of learners are applied to the unlabeled data to generate pseudo-labels which are used for training in the next iterations. Pseudo-labels, $l(x_i)$, $k<i<k+u$, are simply the most confident labels produced by the learner or set of learners for a set of unlabeled samples, $\mathcal {X}_U \subset \mathcal {D}_U$, [31]. The wrapper methods we will consider are self-training and co-training. According to the way they make use of the unlabeled data, unsupervised preprocessing methods can be divided into feature extraction, unsupervised clustering and parameter initialization or pre-training.

*2.1.1.1.1 Wrapper methods* In wrapper methods, a model is first trained on labelled data to generate pseudo-labels for an unlabeled subset, $\mathcal {X}_U \subset \mathcal {D}_U$, then the model is iteratively re-trained, until all unlabeled data are labelled or some stopping criterion is met, with a new dataset containing both the labelled dataset, $\mathcal {D}_L$, and the pseudo-labels, $l{(x_i)}$, $k<i<k+u$, of the subset $\mathcal {X}_U$, generated in previous iterations. They are the most well-known and oldest SSL methods [27, 31]. Wrapper methods may be used for classifictaion and regression and are divided into three categories: self-training, co-training, and boosting.

1. Self-training. Self-training [32] also referred to as self-learning, are wrapper methods that consist of a single base SL learner that is iteratively trained on a training set consisting of the original labelled data and the high-confidence predictions, pseudo-label, from the previous iterations. They are the most basic wrapper methods [31] and may be applied to most, if not all, SL algorithms such as Random Forests (RF) [33], Support Vector Machines (SVM) [34], etc.
2. Co-training. Co-training methods, [35, 36], assume that (i) features can be split into two or more distinct sets or views; (ii) each feature subset is sufficient to train a good classifier; (iii) the views are conditionally independent given the class label. Co-training extends the principle of self-training to multiple SL learners that are each iteratively trained with the pseudo-labels from the other learners, in other words, learners “teach” each other with the added pseudo-labels to improve global performance. For co-training to work well, the sufficiency (ii) and independence (iii) assumptions should be satisfied [35]. Multi-view co-training, the basic form of co-training, constructs two learners on distinct feature sets or views. When no natural feature split is known a priori, single-view co-training may be used to build two or more weak learners with different hyper-parameters on the same feature set. There exist several approaches based on single-view co-training such as tri-training [37], co-forest [38], co-regularization [39], etc. In co-regularization, the two terms of the objective function minimize the error rate and optimize the disagreement between base learners [39].

*2.1.1.1.2 Unsupervised preprocessing* The unsupervised preprocessing methods use $\mathcal {D}_U$ and $\mathcal {D}_L$ at two different steps. The first step often consists of extraction (feature extraction) or transformation (unsupervised clustering) of the feature space or for initialization of a model’s parameters (pre-training) while the second step consists of using knowledge from $\mathcal {D}_L$ to label the unlabeled data points in $\mathcal {D}_U$. We briefly describe the methods in the next points.

1. Feature Extraction: Feature extraction is one of the most critical steps to take in ML. It consists of extracting a set of relevant features for ML models to work. Typically, SSL feature extraction methods consist of either finding lower-dimensional feature spaces, from $\mathcal {X}$, without sacrificing significant amounts of information or finding lower-dimensional vector representations of highly dimensional data objects by considering the relationships between the inputs. Examples of SSL feature extraction methods are autoencoder (AE) [14] and a few of its variants, such as denoising autoencoder [40] and contractive autoencoder [41], and methods in NLP (Natural Language Processing) such as Word2Vec [42], GloVe [43], etc.
2. Unsupervised clustering: Also referred to as cluster-then-label methods, these methods explicitly join the SL or SSL classification or regression algorithms and UL or SSL clustering algorithms. The UL or SSL clustering algorithm first clusters all the data points, then those clusters are fed to the SL or SSL classifier or regressor for label inference [44–46].

*2.1.1.1.3 Intrinsically semi-supervised* Intrinsically semi-supervised methods are typically extensions of existing SL methods to directly include the information from unlabeled data points in the loss function. Regarding the SSL assumption they rely on, these methods can be further grouped into four categories: (i) maximum-margin methods, where the goal is to maximize the distance between data points and the decision boundary (low density-assumption), (ii) perturbation-based methods, often implemented with neural networks (NN), rely directly on the smoothness assumption (a noisy, or perturbated, version of a data point should have the same predicted label, as the original data point), (iii) manifold-based methods either explicitly or implicitly estimate the manifolds on which the data points lie and (iv) generative models whose primary goal is to infer a function that can generate samples, similar to the available samples, from random noise.

##### Transductive methods

A learner is said to be transductive if it only works on the labelled and unlabeled data available at training and cannot handle unseen data [17]. The goal of a transductive learner is to infer labels for an unlabeled dataset $\mathcal {D}_U$, using $\mathcal {D}_L$. If a new unlabeled data point, $x_u \notin \mathcal {D}_U$, is given, the learner must be reapplied, from scratch to all the data, i.e., $\mathcal {D}_L$, $\mathcal {D}_U$, and $x_u$. Graph-based methods, which are often transductive in nature, define a graph where the nodes are labelled and unlabeled samples in the dataset, and edges (weighted) reflect the similarity of the samples. These methods usually assume label smoothness over the graph. Graph methods are non-parametric and discriminative [17]. The defined loss function is optimized to achieve two goals: (i) for already labelled samples, from $\mathcal {D}_L$, the inferred labels should correspond to their true labels and (ii) the predicted labels of similar samples on the graph be the same. A transductive learner’s task may be classification or regression.

#### SSL for clustering

Semi-supervised clustering methods can be used with partially labelled data as well as other types of outcome measures. When cluster assignments, or partial labels, for a subset of the data, are known beforehand, the objective is to classify the unlabeled samples using the known cluster assignments [47], this is, in a sense, equivalent to an SL problem. When more complex relationships among the samples are known in the form of constraints, the problem becomes a generalization of the previous objective and is either called constrained clustering [48], i.e., an existing clustering method is modified to satisfy the constraints, or distance-based (metric-based) clustering, i.e., an alternative distance metric is used to satisfy the constraints [49, 50].

Hierarchical and partitional clustering techniques are the two main types of clustering algorithms. Hierarchical clustering methods recursively locate nested clusters in either agglomerative or divisive mode. In agglomerative mode, they start with each data point in its own cluster and merge the most similar clusters successively to form a cluster hierarchy and in divisive or top-down mode, they start with all the data points in one cluster and recursively divide each cluster into smaller clusters [51]. SS Hierarchical clustering methods group samples using a tree-like architecture, known as a hierarchy. They either built separate hierarchies for must-link and cannot-link constrained samples [52–55] or use other types of constraints [56–60]. Finally, SS Clustering may be used to build clusters related to a given outcome variable [61].

We refer the interested reader to [16, 25, 26, 29, 62] for detailed descriptions of the methods mentioned in this section.

### Cybercrimes

As mentioned in Sect. 1, a cyber-attack is any offensive maneuver that targets computer systems aiming at information disclosure, theft of or damage to their hardware, software, or electronic data, as well as from the disruption or misdirection of the services they provide, and cyber-security can be defined as the protection of computer systems against cyber-attacks [19]. Cybercrimes are criminal activities that involve the use of digital technologies such as computers, smartphones, the internet, and other digital devices [63]. From a legal perspective, cybercrimes can be defined as criminal offences that involve the use of a computer or a computer network [64]. The cyber-attacks covered in this article can all be seen as specific types of cybercrime, we, therefore, use the two terms interchangeably. Note that different jurisdictions may have different laws regarding what constitutes a cybercrime or cyber-attack. Therefore, an activity that is considered a cyber-attack in one jurisdiction may not be considered a cybercrime in another jurisdiction, depending on the specific laws in each location but cybercrimes typically involve the illegal or unauthorized use of digital technologies such as computers [63, 64]. Additionally, some activities that are not considered cyber attacks in some jurisdictions may still be considered cybercrimes if they violate specific laws related to computer systems and networks [63]. Cybercrimes may also be viewed from technical [65] and procedural [66, 67] perspectives.

The IBM X-Force Incident Response and Intelligence Services (IRIS) estimated the profit made by a group of attackers to be over US$123 million in 2020 [68] and the Cost of a Data Breach report published in 2021 by IBM Security estimates the global average cost per incident to US$4.24 million [69]. Cybercriminals are always taking advantage of catastrophes, disasters, and hot events for their own gains. A clear example is the surge in cybercrimes of all sorts witnessed at the beginning of the pandemic.

The following subsections briefly describe the cybercrimes countered in the covered literature.

#### Network intrusion

Any unlawful action on a digital network is referred to as network intrusion. Network intrusions or breaches can be thought of as a succession of acts carried out one after the other, each dependent on the success of the last. The stages of the intrusion are sequential, beginning with reconnaissance and ending with the compromising of sensitive data [70]. These principles are useful for managing proactive measures and finding bad actors’ behaviour. Network intrusions often include the theft of valuable network resources and virtually always compromise network and/or data security [71, 72]. Living off the land, multi-routing, buffer overwriting, covert CGI scripts, protocol-specific attacks, traffic flooding, Trojan horse malware, and worms are the most frequent intrusion attacks.

Some intruders will attempt to implant code that cracks passwords, logs keystrokes, or imitates a website in order to lead unaware users to their own. Others will infiltrate the network and steal data on a regular basis or alter websites accessible to the public with a range of messages. Intruders may get access to a computer system in a number of ways, including internally, externally, or even physically.

#### Phishing

IBM X-Force identified phishing as one of the most used attack vectors in 2021 because of their ease of use and low resource requirements [73]. Phishing is a form of cybercrime where the attackers’ aim is to trick users into revealing sensitive data, including personal information, banking, and credit card details, IDs, passwords, and more valuable information via replicas of legitimate websites of trusted organizations. Phishing attacks can be grouped into deceptive phishing and technical subterfuge [74]. Deceptive phishing is often performed via emails, SMS, calendar invitations, using telephony, etc., and technical subterfuge is the act of tricking individuals into disclosing their sensitive information through technical subterfuge by downloading malicious code into the victim’s system. We refer the reader to a recent in-depth study on phishing attacks [74].

#### Spam

Spam, not to be mistaken for canned meat, may be defined as unsolicited and unwanted messages, typically sent in bulk, that can take several forms such as email, text messages, phone calls, or social media messages. The content of spam messages can vary widely, but they are often commercial in nature and aim to advertise a product or service or promote a fraudulent scheme or solicit donations [75].

#### Malware

Malware or malicious software is defined as any software that intentionally executes malicious payloads on victim machines (computers, smartphones, computer networks, and so on) to cause disruptions. There exist several varieties of malware, such as computer viruses, worms, Trojan horses, ransomware, spyware, adware, rogue software, wipers, and scareware. In the 2022 Threat Intelligence Index, IBM X-Force reported that ransomware, a type of malware, was again the top attack type in 2021, although decreasing from 23%, in 2020, to 21% [73]. Defensive tactics vary depending on the type of malware, but most may be avoided by installing antivirus software and firewalls, applying regular patches to decrease zero-day threats, safeguarding networks from intrusion, performing regular backups, and isolating infected devices.

#### Other cyber-attacks

In addition to intrusions, spam, phishing and malware, we also discuss SSL applications for:

1. *Traffic classification* - traffic classification may be used to detect patterns suggestive of denial-of-service attacks, prompt automated re-allocation of network resources for priority customers, or identify customer use of network resources that in some manner violates the operator’s terms of service [76];
2. *Sybil detection*—a Sybil attack may be defined as an attack against identity in which an individual entity masquerades as numerous identities at the same time [77];
3. *Stock market manipulation detection*—market manipulation may be defined as an illegal practice in an attempt to boost or reduce stock prices by generating an illusion of an active trading [78, 79];
4. *Social bot detection*—a social bot may be defined as a social media account that is operated by a computer algorithm to automatically generate content and interact with humans (or other bot users) on social media, in an attempt to mimic and possibly modify their behaviour [80, 81];
5. *Shilling attack detection*—a Shilling attack is a particular type of attack in which a malicious user profile is injected into an existing collaborative filtering dataset to influence the recommender system’s outcome. The injected profiles explicitly rate items in a way that either promotes or demotes the target items [82];
6. *Pathogenic social media account detection*—Pathogenic Social Media (PSM) accounts refer to accounts that have the capability to spread harmful misinformation on social media to viral proportions. Terrorist supporters, water armies, and fake news writers are among the accounts in this category [83, 84];
7. *Fraud detection*—in the banking industry such as credit card fraud detection. Credit card fraud may happen when unauthorized individuals obtain access to a person’s credit card information and use it to make purchases, other transactions, or open new accounts [85]; and
8. *Detection of attacks on other platforms* such as the power grid - the smart grid enables energy customers and providers to manage and generate electricity more effectively. The smart grid, like other emerging technology, raises new security issues [86].

### Examples of industry deployments of ML in cyber-security

This section presents examples of successful industrial deployments of ML for countering cyber threats. The first example is “IBM X-Force Threat Management” [87], an ML platform deployed to counter cyber threats. IBM X-Force Threat Management is a cloud-based security platform that leverages ML to provide advanced threat detection and response capabilities. It analyzes massive amounts of security data, including network traffic, system logs, and user behaviour, to identify and respond to potential threats in real-time using ML algorithms. The ML models are trained on large datasets of historical security events, allowing the system to learn and adapt to new threats over time. Depending on the use case and data available, it is possible that IBM X-Force Threat Management may use a combination of ML techniques, such as SSL and Reinforcement Learning, in addition to other optimization methods for enhancing security policies. However, it should be noted that without specific information from IBM, it cannot be definitively confirmed whether these techniques are actually employed. Nonetheless, the platform has demonstrated success in detecting various types of cyber threats, including banking Trojans such as IcedID,^1^ TrickBot and QakBot.

The second example is the Deep Packet Inspection (DPI) system developed by Darktrace, a cyber-security company. The system uses unsupervised ML algorithms to learn the expected behaviour of a network and detect anomalies that may indicate malicious activity. The system can also automatically respond to detected threats by initiating a range of actions, such as quarantining a device or blocking network traffic. Darktrace has deployed its DPI system in various industries, including healthcare, finance, and energy. In one instance, a UK construction company used the system to detect and respond to a ransomware attack.^2^ The system identified the attack within minutes of it starting and initiated a range of responses, including blocking the attacker’s IP address and quarantining affected devices. The company was able to contain the attack and avoid paying the ransom demanded by the attackers.

Our third example is Feedzai, an ML platform that provides fraud prevention and anti-money laundering for financial institutions and businesses. Feedzai employs a variety of ML techniques, including Deep Learning and combining SL and UL (SSL),^3^ to detect and prevent fraudulent activity in real-time. After partnering with a large European bank, Feedzai’s platform reduced false positives and accurately identified fraudulent activity, resulting in lower losses due to fraud.^4^

Overall, IBM X-Force Threat Management, Darktrace, and Feedzai demonstrate how ML can be successfully deployed in the industry to counter cyber threats and provide advanced threat detection and response capabilities.

## Review methodology

This section provides the details of the methodology we followed. To achieve our goal of reviewing the datasets and evaluation metrics used in the applications of SSL techniques to cyber-security, we followed the standard systematic literature review guidelines outlined in [88] for assessing the search’s completeness. The entire process was done on Covidence [89], an online tool for systematic review management and production. We first defined our three research questions shown below. These are motivated by the need to examine the efforts being made to safeguard users and computer systems against attacks using SSL. This stems from the fact that attacks are far more harmful than vulnerability scans or related operations. We intend to review the datasets as well as the evaluation metrics used in the literature identifying the cyber-attacks as soon as possible to take the necessary actions to reverse them.

1. With the introduction and use of SSL in cyber-security, what are the assessment metrics used to evaluate the built models?
2. What datasets are the proposed SSL approaches built upon? What are the most used datasets?
3. What are the open challenges with respect to the datasets and performance assessment metrics?

Our inclusion and exclusion criteria were then defined from the above research questions. A paper is included if it directly applies SSL for detecting at least one of the cyber-attacks mentioned in Sect. 2.2. with enough details to address our research questions. On the other hand, a paper is excluded if (i) another paper of the same authors superseded the work, in which case the latest work is considered, (ii) it does not use SSL for the inclusion criteria and (iii) the approach is discussed at a high level, with insufficient information to fulfill the research questions. The entire process was done on Covidence [89], an online tool for systematic review management and production. We then queried IEEE Xplore and ACM Digital Library for articles having (“semi-supervised learning” AND “cyber-security”), (“semi-supervised” AND “cyber-security”) and (“semi-supervised” AND “security”) anywhere within the article.

The keywords (“semi-supervised learning” AND “cyber-security”) have been chosen because SSL has been increasingly used in cyber-security to improve the accuracy of detection and classification systems [90]. This combination has been used to find articles that specifically focus on using SSL in cyber-security tasks such as intrusion detection, malware detection, network traffic analysis, etc. Similarly, the combination of (“semi-supervised” AND “cyber-security”) has been used to find articles that discuss semi-supervised learning in a cyber-security context, even if they do not explicitly mention the phrase “semi-supervised learning”. Finally, the combination (“semi-supervised” AND “security”) has been used to broaden the search beyond just cyber-security and potentially include other domains where SSL has been applied to security-related tasks.

Note that we did not limit the search to the title, abstract or keywords because it was essential to making sure to find all the articles discussing and applying SSL methods for cyber-security for screening. The reason we chose these databases is that they are among the top databases suggested by our university library for conducting Computer Science research and they also contain papers published in top-tier venues. To complement the results obtained from IEEE Xplore and ACM Digital Library, we submitted the same search queries to Google Scholar and extracted the top 200 search results sorted by relevance. The combinations mentioned earlier and this search strategy allowed us to find articles that are relevant to using SSL in cyber-security, and gain a better understanding of how it is being/has been used to improve security systems.

**Fig. 2:**
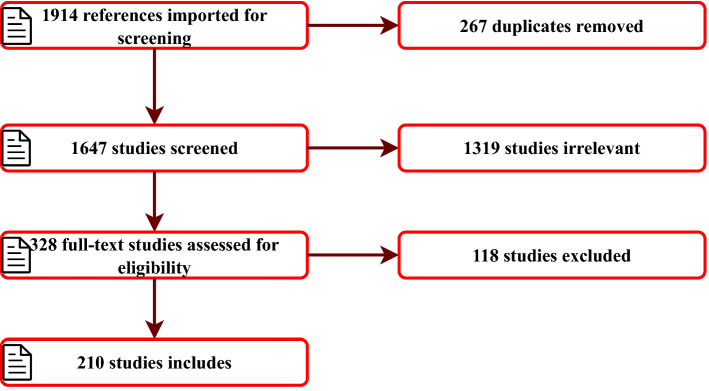
Review methodology

As seen in Fig. 2, in total, 1914 studies were imported for screening; 267 duplicates were automatically removed, and the remaining 1647 studies’ titles and abstracts were manually screened for relevance. Based on our inclusion and exclusion criteria, 1319 studies were found irrelevant, because they either did not discuss SSL methods or cyber-attack defences. The remaining 328 studies’ full texts were further assessed as they were either partially or fully related to our inclusion criteria, and finally, 210 relevant studies were included for data extraction. Furthermore, we used state-of-the-art surveys and review articles on SSL [16, 27] and ML for cyber-security [4] to construct this extensive review of cyber-security datasets and performance evaluation metrics for SSL models.

## Datasets and performance assessment metrics

In this section, we summarize and analyze the public datasets and performance assessment metrics used in the selected papers.

### Datasets and repositories

**Fig. 3:**
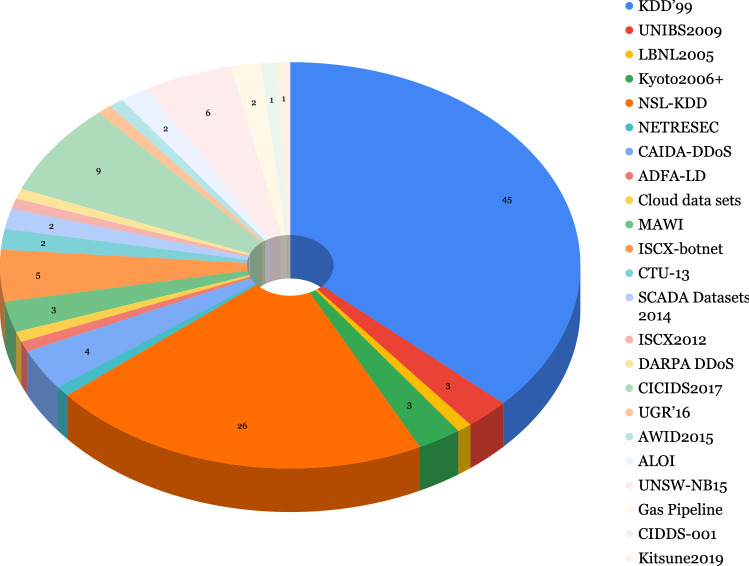
Usage of intrusion detection datasets and sources in selected papers

**Fig. 4:**
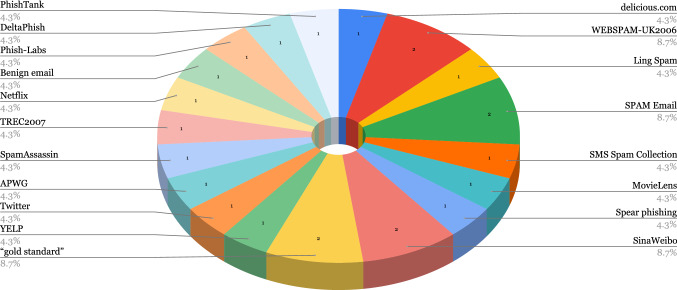
Usage of spam and phishing datasets and sources in selected papers

**Fig. 5:**
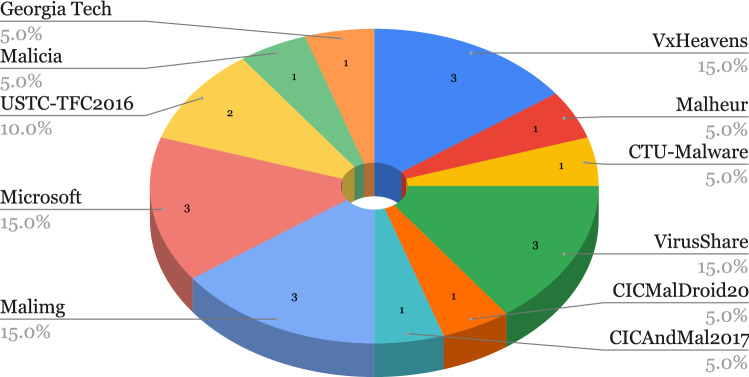
Usage of malware datasets and sources in selected papers

**Fig. 6:**
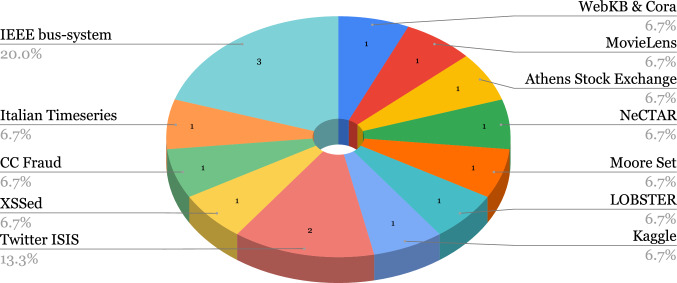
Usage of additional datasets and sources in selected papers

AI, especially ML, has proven itself a particularly useful tool in cyber-security as well as other fields of computer science and has extensively featured in the literature for cybercrime or malicious activity detection. “Cost of a Data Breach” [69], published by IBM Security, reported a US$3.81 million, or almost 80% difference between breach costs of companies with fully deployed security AI/ML and automation and companies without security AI/ML and automation. We present the public datasets used in the covered literature in this section, grouped by type of attack and show their usage in the selected papers in Figs. 3, 4,  5, and  6. Note that we acknowledge the difference between Spam and Phishing in Subsections 2.2.3 and 2.2.2 as they are different attack vectors but due to the scarcity of these datasets, we have combined them in a single section.

#### Network intrusion datasets and sources

In terms of network intrusion, we found a total of 18 public datasets and sources in the papers we reviewed. We begin by providing a brief description of each dataset; we, then, provide a summary of their main characteristics as well as some key data usage statistics.

1. *KDD’99 and NSL-KDD*. The KDD’99 dataset is a statistically preprocessed dataset which has been available since 1999 from DARPA [91], it is an updated version of the DARPA98. It is the most used dataset in the selected papers. The dataset has three components, basic, content and traffic features, making a total of 41 features for normal and simulated attack traffic. The NSL-KDD dataset, proposed by Tavallaee [92], is a version of the KDD’99 dataset in which redundant records are removed to enable the classifiers to produce unbiased results. The two datasets contain various attack types such as Neptune-DoS, pod-DoS, Smurf-DoS, and buffer-overflow. Table 1 gives a brief composition of the KDD’99 and NSL-KDD datasets.
2. *Moore Set*. The Moore Set [93] was prepared in 2005 by researchers at Intel Research. It comprises real-world traces collected by the high-performance network monitor. Each object in the Moore set represents a single flow of TCP packets between client and server, which consists of 248 characteristics. The information in the features is derived using packet header information alone, while the classification- class has been derived using content-based analysis. Table 2 shows a brief composition of the Moore Set.
3. *LBNL2005.* The Lawrence Berkeley National Laboratory (LBNL) 2005 traffic traces were collected at the LBNL/ICSI under the Enterprise Tracing Project over a period of three months in 2004 and 2005 on two routers [94]. It contains full header network traffic recorded at a medium-sized enterprise covering 22 subnets and includes trace data for a wide range of traffic including web, email, backup, and streaming media. Because the traffic traces are completely anonymized, all the packets do not have a payload. As seen in Table 3, the LBNL trace consists of five datasets labelled: D0–D4. The “Per Tap” row specifies the number of traces collected on each monitored router port while the “Snaplen” row gives the maximum number of bytes recorded for each packet.
4. *CAIDA Datasets*. The Centre for Applied Internet Data Analysis (CAIDA), based at the University of California’s San Diego Supercomputer Center, collects a variety of data from geographically and topologically diverse locations and makes it available to the research community to the extent possible while respecting the privacy of individuals and organizations who donate data or network access. The CAIDA-DDoS Dataset [95], comprises approximately one hour of anonymized traffic from a DDoS attack on August 4, 2007 (20:50:08 UTC to 21:56:16 UTC). This type of denial-of-service attack tries to prevent access to the targeted server by using all of the server’s computational power and all of the bandwidth on the network linking the server to the Internet. The traces only include attack traffic to the victim and responses to the attack from the victim. Non-attack traffic has been eliminated to the greatest extent practicable.
5. *Kyoto2006+*. The Kyoto2006+ is a publicly available benchmark dataset, consisting of 24 statistical features, that is built on three years of network traffic, from November 2006 to August 2009 [96]. It covers both regular servers and honeypots deployed at Kyoto University in Japan labelled as normal (no attack), attack (known attack) and unknown attack. It includes a variety of attacks performed against the honeypots such as shellcode, exploits, DoS, port scans, backscatter, and malware, shown in Table 4. An updated version of the dataset contains additional data collected from November 2006 to December 2015 [97].
6. *UNIBS2009*. The UNIBS-2009 trace [98], was compiled by the University of Brescia in 2009. It consists of traffic traces collected by running Tcpdump on the edge router of the university’s campus network on three consecutive working days (2009.9.30, 2009.10.1 and 2009.10.02) connecting the network to the Internet through a 100 Mbps uplink. As shown in Table 5, the dataset supplies the true labels, and the traffic trace includes Web (HTTP and HTTPS), Mail (POP3, IMAP4, SMTP and their Secure Sockets Layer variants), Skype, P2P (BitTorrent, Edonkey), SSH (Secure Shell), FTP (File Transfer Protocol) and MSN.
7. *UNB ISCX-2012*. The Installation Support Center of Expertise (ISCX)-2012 dataset has been prepared at the ISCX at the University of New Brunswick [99]. It is built on 7 days of network traffic, shown in Table 6, and consists of over two million traffic packets characterized by 20 features taking nominal, integer, or float values. The dataset includes full packet payloads in pcap format.
8. *CTU-13*. The CTU-13 dataset was compiled by the Czech Technical University [100]. It consists of botnet traffic captured in the university in 2011. The dataset includes thirteen scenarios, shown in Table 7, covering different botnet attacks, that use a variety of protocols and performing different actions, mixed with normal traffic and background traffic. The dataset is available in the forms of unidirectional flow, bidirectional flow, and packet capture.
9. *SCADA 2014*. The Supervisory Control And Data Acquisition (SCADA) [101] is a database proposed by Mississippi State University Key Infrastructure Protection Center in 2014 to evaluate the industrial network intrusion detection model. It is one of the standard databases in the current industrial control network intrusion detection commonly used in experiments. It includes the Gas system dataset and Water storage system dataset from the Industrial Control System network layer.
10. *UNSW-NB15*. The UNSW-NB15 dataset was compiled in 2015 by the University of New South Wales Canberra at the School of Engineering and IT, UNSW Canberra at ADFA, using a small, emulated network over 31 h by getting normal and malicious raw network packets. It consists of nine attack types: analysis, backdoors, DoS, exploits, generic, fuzzers, reconnaissance, shell code and worms. It consists of over two million records each characterized by 49 features taking nominal, integer, or float values. The dataset’s data distribution is shown in Table 8.
11. *AWID 2015*. The Aegean Wi-Fi Intrusion Dataset (AWID), published in 2015 [102], comprises the largest amount of Wi-Fi network data (normal and attack) collected from real network environments. The 16 attack types can be grouped into flooding, impersonation, and injection. As seen in Table 9, the dataset contains over 5 million samples each characterized by 154 features, representing the WLAN frame fields along with physical layer meta-data.
12. *ISCXVPN2016*. The ISCXVPN2016 [103], published by the UNB in 2016, comprises traffic captured using Wireshark and tcpdump, generating a total amount of 28GB of data. For the VPN, an external VPN service provider connected to using OpenVPN (UDP mode) was used. To generate SFTP and FTPS traffic an external service provider and Filezilla as a client was used. Table 10 shows the data distribution in the ISCXVPN2016 dataset.
13. *CIDDS*. The Coburg Intrusion Detection Datasets (CIDDS), prepared at Coburg University of Applied Sciences (Hochschule Coburg), consist of several labelled flow-based datasets created in virtual environments using OpenStack. The CIDDS database’s most used dataset, CIDDS-001, released in 2017, covers four weeks of unidirectional traffic flows each characterized by 19 features taking nominal, integer, or float values. As seen in Table 11, the dataset includes attacks such as DoS, port scan and SSH brute force.
14. *CICIDS2017*. The Canadian Institute for Cyber-security Intrusion - Evaluation Dataset (CIC-IDS)-2017 was produced in an emulated network environment at the CIC [104]. It is built on 5 days (July 3 to July 7, 2017) of network traffic, shown in Table 12, and includes a variety of most common attack types including FTP patator, SSH patator, DoS slowloris, DoS Slowhttptest, DoS Hulk, DoS GoldenEye, Heartbleed, Brute force, XSS, SQL Injection, Infiltration, Bot, DDoS (Distributed denial of service), and Port Scan each characterized by 80 features extracted using CICFlowMeter [103, 105]. The dataset also includes full packet payloads in pcap format.
15. *UGR’16*. The UGR’16 dataset, proposed in 2018 by Maciá-Fernández et al. [106], comprises NetFlow network traces collected from a real Tier 3 ISP network made up of several organizations’ and clients’ virtualized and hosted services including WordPress, Joomla, email, FTP, etc. NetFlow sensors were installed in the network’s border routers to capture all incoming and outgoing traffic from the ISP. As seen in Table 13, two sets of data are provided: one for training models (calibration set) and the other for testing the models’ outputs (test set).
16. *Kitsune2019*. The Kitsune Network Attack Dataset, Kitsune2019, has been prepared at Ben-Gurion University of the Negev, Israel and was released in May 2018 [107]. The dataset is composed of 9 files covering 9 distinct attacks situations on a commercial IP-based video surveillance system and an IoT network: OS (Operating System) Scan, Fuzzing, Video Injection, ARP Man in the Middle, Active Wiretap, SSDP Flood, SYN DoS, Secure Sockets Layer Renegotiation and Mirai Botnet. It contains 27,170,754 samples each characterized by 115 real features. The violation column in Table 14 indicates the attacker’s security violation on the network’s confidentiality (C), integrity (I), and availability (A).
17. *NETRESEC* is a software company that specializes in network security monitoring and forensics. They also maintain.pcap repository files gathered from various Internet sources [108]. It is a list of freely accessible public packet capture repositories on the Internet. Most of the websites listed on their website provide Full Packet Capture (FPC) files, however, others only provide truncated frames.
18. *MAWI archive*. The MAWI archive [109] consists of an ongoing collection of daily Internet traffic traces captured within the WIDE backbone network at several sampling points. Tcpdump is used to retrieve traffic traces, and the IP (Internet Protocol) addresses in the traces are encrypted using a modified version of Tcpdpriv (MAWI Working Group Traffic Archive (http://www.wide.ad.jp)). The samplepoint-F consists of daily traces at the transit link of WIDE to the upstream ISP and has been in operation since 01/07/2006.
19. *Kaggle*^5^ is an online data sharing and publishing platform. It includes security-based datasets such as KDD’99 and NSL-KDD. Registered users can also upload and explore data analysis models.

**Table 1:** KDD’99 and NSL-KDD composition

	KDD’99 composition		NSL-KDD composition	
	Train samples	Test samples	Train samples	Test samples
Attacks	3,925,650	250,436	58,630	9083
Normal	972,781	60,591	67,343	9711
Total	4,898,431	311,027	125,973	18,794

**Table 2:** Moore set composition

Category	Number of flows
WWW	328,092
MAIL	28,567
FTP-CONTROL	3054
FTP-PASV	2688
ATTACK	1793
P2P	2094
DATABASE	2648
FTP-DATA	5797
MULTIMEDIA	576
SERVICES	2099
INTERACTIVE	110
GAMES	8
TOTAL	377,526

**Table 3:** LBNL2005 composition

	D0	D1	D2	D3	D4
Date	4/10/2004	15/12/2004	16/12/2004	6/1/2005	7/1/2005
Duration	10 min	1 h	1 h	1 h	1 h
Per Tap	1	2	1	1	1–2
# Subnets	22	22	22	18	18
# Packets	17.8 M	64.7 M	28.1 M	21.6 M	27.7 M
Snaplen	1500	68	68	1500	1500
Mon. Hosts	2531	2102	2088	1561	1558
LBNL Hosts	4767	5761	5210	5234	5698
Remote Hosts	4342	10,478	7138	16,404	23,267

**Table 4:** Kyoto2006+ composition

Month	Normal flows	Attack flows	Total flows
January	2,216,820	1,453,356	3,670,176
February	1,947,619	1,373,913	3,321,532
March	1,861,103	1,537,011	3,398,114
April	2,637,097	1,130,039	3,767,136
May	2,153,355	1,594,172	3,747,527
June	1,852,554	1,891,101	3,743,655
July	2,086,698	1,784,027	3,870,725
August	1,640,651	2,070,290	3,710,941

**Table 5:** UNIBS2009 composition

Class of protocols	Flows	Bytes
Web	61.2%	12.5%
Mail	5.7%	0.2%
P2P (BitTorrent)	9.3%	15.9%
P2P (Edonkey)	18.4%	70.2%
Skype (TCP (Transmission Control Protocol))	1.4%	1.0%
Skype (UDP (User Datagram Port))	3.8%	0.0%
Other	0.2%	0.2%
Total	78,998	27 GB

**Table 6:** ISCX-2012 composition

Day	Date	Description	Size (GB)
Friday	11/06/2010	Normal activity. No malicious activity	16.1
Saturday	12/06/2010	Normal activity. No malicious activity	4.22
Sunday	13/06/2010	Infiltrating the network from Inside + normal activity	3.95
Monday	14/06/2010	HTTP Denial of Service + Normal activity	6.85
Tuesday	15/06/2010	DDoS using an IRC Botnet	23.4
Wednesday	16/06/2010	Normal activity. No malicious activity	17.6
Thursday	17/06/2010	Brute Force SSH + Normal Activity	12.3

**Table 7:** CTU-13 composition

Scen.	Total flows	Botnet	Normal	C &C	Background
1	2,824,636	39,933 (1.41%)	30,387 (1.07%)	1026 (0.03%)	2,753,290 (97.47%)
2	1,808,122	18,839 (1.04%)	9120 (0.5%)	2102 (0.11%)	1,778,061 (98.33%)
3	4,710,638	26,759 (0.565)	116,887 (2.48%)	63 (0.001%)	4,566,929 (96.94%)
4	1,121,076	1719 (0.15%)	25,268 (2.25%)	49 (0.004%)	1,094,040 (97.58%)
5	129,832	695 (0.53%)	4,6794 (3.6%)	206 (1.15%)	124,252 (95.7%)
6	558,919	4431 (0.79%)	7,494 (1.34%)	199 (0.03%)	546,795 (97.83%)
7	114,077	37 (0.03%)	1,677 (1.47%)	26 (0.02%)	112,337 (98.47%)
8	2,954,230	5052 (0.17%)	72,822 (2.46%)	1074 (2.4%)	2,875,2821 (97.32%)
9	2,753,884	179,880 (6.5%)	43,340 (1.57%)	5099 (0.18%)	2,525,565 (91.7%)
10	1,309,791	106,315 (8.11%)	15,847 (1.2%)	37 (0.002%)	1,187,592 (90.67%)
11	107,251	8161 (7.6%)	2718 (2.53%)	3 (0.002%)	96,369 (89.85%)
12	325,471	2143 (0.65%)	7628 (2.34%)	25 (0.007%)	315,675 (96,99%)
13	1,925,149	38,791 (2.01%)	31,939 (1.65%)	1202 (0,06%)	1,853,2171 (96.26%)

**Table 8:** UNSW-NB15 Composition

Class	Train set		Test set	
	No. of Dup/ Dup %	No. of Rec	No. of Dup/ Dup %	No. of Rec
Normal	4110/7.33	56,000	0/0.00	37,000
Fuzzers	2034/11.18	18,184	0/0.00	6062
Analysis	405/20.25	2000	0/0.00	677
Backdoors	211/12.08	1746	0/0.00	583
DoS	8457/68.95	12,264	0/0.00	4089
Exploits	13,548/40.57	33,393	0/0.00	11,132
Generic	35,819/89.54	40,000	0/0.00	18,871
Reconnaissance	2969/28.30	10,491	0/0.00	3496
Shellcode	42/3.70	1133	0/0.00	378
Worms	3/2.30	130	0/0.00	44
All	74,072/42.24	175,341	0/0.00	82,332

*Dup* Duplicates, *Rec* Records

**Table 9:** AWID 2015 composition

AWID-ATK-F-TRn		AWID-ATK-F-Tst	
12,416	amok	3856	amok
1,529,284	arp	500,823	arp
93,011	auth_req	34,833	auth_req
170,826	beacon	5498	beacon
1,860,780	cafe_latte	16,719	cafe_latte
817,954	deauth	22,879	chop_chop
23,598	evil_twin	38,359	cts
1098	frag	33,870	deauth
157,749,037	normal	34,871	disassociation
117,252	probe_response	27,045	evil_twin
		240	frag
		433,750	hirte
		47,325,477	Normal
		13,551	power
		10,981	probe_request
		8578	probe_response
		13,536	rts

AWID-CLS-F-Trn		AWID-CLS-F-Tst	
1,211,459	flooding	197,933	flooding
1,884,378	impersonation	477,514	impersonation
1,530,373	injection	523,942	injection
157,749,037	normal	47,325,477	normal

**Table 10:** ISCXVPN2016 composition

Traffic	Content	Number	Percentage
Email	POP3, SMTP and IMAP	26,844	14.94%
Chat	ICQ, Skype, AIM, Hangouts and Facebook	33,978	18.92%
Streaming File	YouTube and Vimeo	26,682	14.85%
Transfer	SFTP, FTPS and Skype using Filezilla	30,000	16.70%
VoIP	Facebook, Skype and Hangouts voice calls (1 h duration)	30,000	16.70%
P2P	Transmission (BitTorrent) and uTorrent	32,130	17.89%

**Table 11:** CIDDS-001 composition

Category	Train set	Test set
Normal	4032	1218
Suspicious	62,539	19,567
Unknown	21,772	6734
Attacker	5888	1890
Victim	3705	1197
Total	97,936	30,606

**Table 12:** CICIDS2017 composition

Days	Labels	Size (GB)
Monday	Benign	11.0
Tuesday	BForce, SFTP, SSH and benign	11
Wednesday	DoS and Heartbleed Attacks slowloris, Slowhttptest, Hulk, GoldenEye and benign	13
Thursday	Web and Infiltration Attacks Web BForce, XSS and Sql Inject. Infiltration Dropbox Download, Cool disk and benign	7.8
Friday	DDoS LOIT, Botnet ARES, PortScans (sS, sT, sF, sX, sN, sP, sV, sU, sO, sA, sW, sR, sL and B) and benign	8.3

**Table 13:** UGR’16 composition

Feature	Calibration	Test
Capture start	10:47 h 03/18/2016	13:38 h 07/27/2016
Capture end	18:27 h 06/26/2016	09:27 h 08/29/2016
Attacks start	N/A	00:00 h 07/28/2016
Attacks end	N/A	12:00 h 08/09/2016
Number of files	17	6
Size (compressed)	181GB	55GB
# Connections	$\approx$ 13,000 M	$\approx$ 3,900 M

**Table 14:** Kitsune2019 composition

Attack type	Attack name	Tool	Description: The attacker	Violation	# Packets
Reconnaissance	OS Scan	Nmap	...scans the network for hosts, and their operating systems, to reveal possible vulnerabilities.	C	1,697,851
	Fuzzing	SFuzz	...searches for vulnerabilities in the camera’s web servers by sending random commands to their cgis.	C	2,244,139
Man in the Middle	Video Injection	Video Jack	...injects a recorded video clip into a live video stream.	C, I	2,472,401
	ARP MitM	Ettercap	...intercepts all LAN traffic via an ARP poisoning attack.	C	2,504,267
	Active Wiretap	Raspberry PI 3B	...intercepts all LAN traffic via an active wiretap (network bridge) covertly installed on an exposed cable.	C	4,554,925
Denial of Service	SSDP Flood	Saddam	...overloads the DVR by causing cameras to spam the server with UPnP advertisements.	A	4,077,266
	SYN DoS	Hping3	...disables a camera’s video stream by overloading its web server.	A	2,771,276
	Secure Sockets Layer Renegotiation (SSLR)	THC	...disables a camera’s video stream by sending many SSLR packets to the camera.	A	6,084,492
Botnet Malware	Mirai	Telnet	...infects IoT with the Mirai malware by exploiting default credentials, and then scans for new vulnerable victim networks.	C, I	764,137

A breakdown of the usage of the Intrusion Detection datasets in the selected papers is shown in Fig. 3, we also provide an overview of the Network Intrusion datasets in Table 15. As seen in Fig. 3, the KDD’99 dataset, despite being old and containing redundant and noisy records, is the most used of the 17 intrusion detection datasets described in this section. 45 out of the 100 selected papers used either the KDD’99 alone or in conjunction with some other intrusion detection dataset. This dataset is followed by the NSL-KDD dataset which is only a smaller version without the redundant and noisy records present in KDD’99. Additionally, none of these datasets are balanced, therefore suitable evaluation metrics should be used when evaluating models built on these datasets. We must highlight that the four most recent datasets used in the papers reviewed were already published in 2017 and 2018 and they have not been extensively explored in an SSL context. Finally, we refer the interested reader to a recent comprehensive survey of Network-based Intrusion datasets [2].

**Table 15:** Overview of network intrusion datasets

Dataset	Pub. year	Size	No of features	Anon.?	Predef. Splits?	Balanced?	Labelled?
KDD’99	2000	5 M Sps	41	No	Yes	No	Yes
LBNL- 2005	2005	160 M Pkts	n.s	Yes	No	No	No
Moore Set	2005	377k Fls	248	No	No	No	Yes
CAIDA- DDoS	2007	n.s	n.s	Yes	No	n.s	No
Kyoto- 2006+	2009	93 M Sps	24	Yes	No	No	Yes
NSL- KDD	2009	150k Sps	41	No	Yes	No	Yes
UNIBS- 2009	2009	79k Fls	n.i.a	Yes	Yes	No	No
ISCX- 2012	2012	2 M Pkts	20	No	No	No	Yes
CTU- 13	2013	81 M Fls	n.i.a	Yes	No	No	Yes
AWID- 2015	2015	5 M Sps	154	No	Yes	No	Yes
UNSW- NB15	2015	2 M Sps	49	No	Yes	No	Yes
ISCX- VPN2016	2016	22 M Fls	8	No	No	No	Yes
CICIDS- 2017	2017	3.1 M Fls	80	No	No	No	Yes
CIDDS- 001	2017	32 M Fls	19	Yes	No	No	Yes
Kitsune- 2019	2018	27 M Sps	115	No	No	No	Yes
UGR’16	2018	16.9B Fls	12	Yes	Yes	No	Yes

n.s.: not specified; n.i.a.: no information available; Fls.: Flows

Anon.: Anonymized; Sps.: Samples; Pkts.: Packets

#### Spam and phishing datasets and sources

1. *Spam Email*. The SPAM Email Dataset contains a total of 4601 emails including 1813 spam emails and 2788 legitimate emails each characterized by 58 attributes. It was donated to the UCI Machine Learning Repository by Hewlett Packard in 1999 [110].
2. *Ling-Spam*. The Ling-Spam dataset, proposed by Androutsopoulos et al. [111] in 2000, contains both spam and legitimate emails retrieved from an email distribution list, the Linguistic list, focusing on linguistic interests around research opportunities, job postings, and software discussion. The dataset contains 2,893 different emails, of which 2,412 are genuine emails collected from the list’s digests and 481 are spam emails retrieved from one of the corpus’ authors.
3. *WEBSPAM-UK2006*. The WEBSPAM-UK2006 dataset was obtained using a set of.UK pages downloaded by the Laboratory of Web Algorithmics of the University of Milan (Università degli Studi di Milano) and manually assessed by a group of volunteers in 2006. The dataset consists of labels, URLs and hyperlinks and HTML page contents of 77,741,046 Web pages [112].
4. *SpamAssassin* (spamassassin.apache.org). Apache SpamAssassin is an Open-Source anti-spam platform providing a filter to classify email and block spam. The SpamAssassin Public mail corpus is a selection of 6,047 emails prepared by SpamAssassin in 2006. Of the total count, there are 1,897 spam messages and 4,150 legitimate emails.
5. *TREC2007 Public Corpus*. The TREC 2007 Public Corpus contains all email messages delivered to a particular server. The server contained several accounts, fallen into disuse and several ‘honeypot’ accounts published on the web, which were used to sign up for a few services, some legitimate and some not. The TREC dataset contains 75,419 messages, of which 25,220 are legitimate emails and 50,199 are junk messages; the messages are divided into three subcorpora [113].
6. *SMS Spam Collection*. The SMS Spam Collection Dataset is a publicly available dataset created by Almeida et al. [114–116] in 2011. It is a labelled dataset of 5574 SMS messages, 747 spam and 4827 ham, collected from mobile phones.
7. *“Gold standard” opinion spam*. The “gold standard” opinion spam dataset was proposed by Ott et al. [117] in 2011. The corpus comprises 1,600 review texts, 800 deceptive and 800 genuine, on 20 hotels in the Chicago area. The genuine reviews were obtained from reviewing websites such as TripAdvisor, Expedia and Yelp and the deceptive ones were rendered using Amazon Mechanical Turk (AMT). In the dataset, 400 reviews are written with a negative sentimental polarity and 400 depict a positive sentimental polarity.
8. *Spear phishing email dataset (2011) & Benign email dataset (2013)*. These two datasets have been prepared by Symantec’s enterprise mail scanning service. The spear phishing email dataset contains 1,467 emails from 8 campaigns and the benign email dataset contains 14,043 emails. The emails were sent between 2011 and 2013, and have attachments, anonymous customer information and PII. The extraction process is described in [118, 119].
9. *MovieLens Dataset*. The GroupLens Research has collected and made available rating datasets from the MovieLens website (https://movielens.org). The datasets were collected over various periods of time, depending on the size of the set. The MovieLens 20 M contains 20 million ratings and 465,000 tag applications applied to 27,000 movies by 138,000 users collected from January 1995 to March 2015 [120].
10. *Netflix*. The Netflix dataset^6^ consists of listings of all the movies and TV shows available on Netflix, along with details such as - cast, directors, ratings, release year, duration, etc.
11. *Twitter* and *Sina Weibo* are two of the most influential social network media platforms in the world. Authors in the selected papers have either used crawlers or APIs to get sample data from these sources.
12. *PhishTank*,^7^**DeltaPhish** [121], **Phish-Labls**^8^ and **Anti-Phishing Working Group**(APWG^9^) are anti-phishing resources that publicly report phishing web pages in an effort to reduce fraud and identity theft caused by phishing and related incidents.
13. *YELP*^10^ and **delicious.com**^11^ publish crowd-sourced reviews about businesses. Similar to Twitter and Sina Weibo, APIs and crawlers may be used to extract data from these sources.

**Table 16:** Overview of spam and phishing datasets

Dataset	Published year	Size	Predef. Splits?	Balanced?	Labelled?
SPAM Email	1999	4601 emails	No	No	Yes
Ling-Spam	2000	2893 emails	No	No	Yes
WEBSPAM UK2006	2006	78 M web pages	No	No	Yes
SpamAssassin	2006	6047 emails	No	No	Yes
TREC2007 Public Corpus	2007	75,419 emails	No	No	Yes
“Gold standard”	2011	1600 samples	No	Yes	Yes
SMS Spam Collection	2011	5574 SMS	No	No	Yes
Spear Phishing Email	2011	1467 emails	No	n/a	n/a
Benign email	2013	14,043 emails	No	n/a	n/a
n/a: not applicable					

A breakdown of the usage of the described Spam and Phishing datasets in the selected papers is shown in Fig. 4, we also provide an overview of the Spam and Phishing datasets in Table 16. We observe that, in the revised works, there is no tendency towards using one or two specific datasets when tackling spam and/or phishing. In effect, the majority of the datasets are used in a single publication and only four, i.e. WEBSPAM-UK2006, Spam Email, SinaWeibo and “gold standard,” out of nineteen are used in two papers as shown in Fig. 4. Additionally, except for the “gold standard” dataset, none of these datasets is balanced.

#### Malware datasets and sources

1. *Georgia Tech Packed-Executable Dataset*. The Georgia Tech Packed-Executable dataset [122] was published in 2008. It consists of 2598 packed viruses collected from the Malfease Project dataset (http://malfease.oarci.net), and 2231 non-packed benign executables collected from a clean installation of Windows XP Home plus several common user applications. The authors also generated 669 packed benign executables by applying 17 different executable packing tools freely available on the Internet to the executables in the Windows XP start menu. Of the 3267 packed executables in their collection, PEiD (http://peid.has.it), one of the most used signature-based detectors for packed executables, was able to detect only 2262 of them, whereas 1005 remained undetected. Therefore, those 1005 undetected samples were kept in the test and the train set contains 4493 samples: 2231 samples related to the non-packed benign executables and 2262 patterns related to the packed executables detected using PEiD.
2. *The Malimg Dataset* [123], proposed in 2011 by the University of California, Santa Barbara, contains 9458 malware images from 25 families.
3. *The Malware Genome Project* [124], proposed by researchers at the North Carolina State University in 2011, contains 1260 Android Malware samples belonging to 49 different malware families collected from August 2010 to October 2011.
4. *Malheur* [125, 126], proposed in 2011, is a tool for the automatic analysis of malware behaviour in a sandbox environment.
5. *Malicia Dataset*. The Malicia dataset [127, 128], published in 2013, comprises 11,688 malware binaries collected from 500 drive-by download servers over a period of 11 months in Windows Portable Executable format. The objective of their work was to identify hosts which spread malware in the wild and to collect samples of malware. In order to collect the samples of malware they set up a honeypot and clients in this honeypot were referring to the malware URL database for downloading and milking the website by resolving the IP address.
6. *CTU-Malware*. The CTU-Malware dataset [129], also compiled by the Czech Technical University, consists of hundreds of captures (called scenarios) of different malware communication samples. Both malware and normal samples are included in the dataset as shown in Table 17.
7. In 2015, Microsoft launched the *Microsoft Malware Classification Challenge*, along with the release of a dataset [130] consisting of over 20,000 malware samples belonging to nine families. Each malware file includes an identifier, which is a 20-character hash value that uniquely identifies the file, and a class label, which is an integer that represents one of the nine families to which the malware may belong.
8. *USTC-TFC2016*. The USTC-TFC2016 dataset [131], published in 2017, consists of ten types of malware traffic from public websites which were collected from a real network environment from 2011 to 2015. Along with such malicious traffic, the benign part contains ten types of normal traffic which were collected using IXIA BPS, a professional network traffic simulation equipment. The dataset’s size is 3.71 GB in the pcap format. The dataset's composition is shown in Table 18.
9. *CICAndMal2017*. The CICAndMal2017 android malware dataset, published in 2018 by the CIC [132], consists of four malware categories namely Adware, Ransomware, Scareware, and SMS Malware and 80 traffic features extracted using CICFlowMeter [103, 105]. The dataset includes 5,065 benign apps from the Google play market published in 2015, 2016, and 2017 and 426 malware samples belonging to 42 unique malware families. The dataset is fully labelled and contains network traffic, logs, API/SYS calls, phone statistics, and memory dumps of malware families shown in Table 19.
10. *CICMalDroid2020*. Also published by the CIC in 2020, the CICMalDroid2020 dataset [133, 134] consists of more than 17,341 Android samples from several sources collected from December 2017 to December 2018. It includes complete capture of static and dynamic features and contains samples spanning between five distinct categories: Adware, Banking malware, SMS malware, Riskware and Benign. Out of 17,341 samples, 13,077 samples ran successfully while the rest failed due to errors such as time-out, invalid APK files, and memory allocation failures. Of the 13,077 samples, 12% failed to be opened mostly due to an “unterminated string” error. From the 11,598 remaining samples, 470 extracted features comprise frequencies of system calls, binders, and composite behaviours, 139 extracted features comprise frequencies of system calls and 50,621 extracted features comprise static information, such as intent actions, permissions, permissions, sensitive APIs, receivers, etc. A brief composition of the dataset is shown in Table 20.
11. *VxHeavens*^12^ is a website dedicated to providing information about malware. The archive comprises over 17,000 programs belonging to 585 malware families (Trojan, viruses, worms).

**Table 17:** CTU-Malware composition

Type	NetFlows	Comm-Pairs
Total	69,180,149	474,884
Normal	13,546,580	445,805
Malware	55,633,569	29,079

**Table 18:** USTC-TFC2016 composition

Benign		Malware		
App type	Size (MB)	Class	Malware type	Size (MB)
Facetime	2.4	Voice/Video	Tinba	2.55
Skype	4.22	Chat/IM	Zeus	13.4
Bittorent	7.33	P2P	Shifu	57.9
Gmail	9.05	Email/Webmail	Neris	90.1
Outlook	11.1	Email/Webmail	Cridex	94.7
WorldOfWarcraft	14.9	Game	Nsisay	281
MySQL	22.3	Database	Geodo	28.8
FTP	60.2	Data transfer	Miuref	16.3
SMB	1206	Data transfer	Virut	109
Weibo	1618	Social Network	Htbot	83.6

*SMB* ServerMessage Block, *IM* Instant Message, *P2P* Peer-to-Peer

**Table 19:** CICAndMal2017 composition

Class	# Families	# Samples
Benign	–	5065
Adware	10	104
Ransomware	10	101
Scareware	11	112
SMS Malware	11	109

**Table 20:** CICMalDroid2020 composition

Type	# Samples
Adware	1253
Banking	2100
SMS malware	3904
Riskware	2546
Benign	1795
Total	11,598

**Table 21:** Overview of malware datasets

Dataset	Pub. year	Size	Predef. Splits?	Balanced?	Labelled?
Georgia Tech	2008	5498 samples	Yes	No	Yes
Malicia	2013	11,688 samples	No	n.s	n.s
CTU-Malware	2015	70 M Flows	No	No	Yes
USTC-TFC2016	2017	752K records	No	No	Yes
CICAndMal2017	2018	5491 samples	n.s	No	Yes
CICMalDroid2020	2020	11,598 samples	No	No	Yes

*n.s.* not specified

We provide an overview of the Malware datasets in Table 21. In Fig. 5, we also show a breakdown of the usage of the described Malware datasets in the selected papers. For these datasets, we observe that out of eleven datasets four have been used in three publications, one was used in two publications and the remaining six have been used only once. In addition, none of these datasets are balanced.

#### Additional datasets and sources

1. *IEEE Test Feeders*. For nearly two decades, the Distribution System Analysis (DSA) Subcommittee’s Test Feeder Working Group (TFWG) has been constructing publicly available distribution test feeders for use by academics. These test feeders aim to create distribution system models that reflect a wide range of design options and analytic issues. The 13-bus and 123-bus Feeders are part of the Test Feeder systems created in 1992 to evaluate and benchmark algorithms in solving unbalanced three-phase radial systems. The DSA Subcommittee approved them during the 2000 Power and Energy Society (PES) Summer Meeting. Schneider et al. [135] summarize the TFWG efforts and intended uses of Test Feeders.
2. *XSSed*^13^ project was created in February 2007. It is an archive of cross-site scripting (XSS) vulnerable websites and provides information on things related to XSS vulnerabilities.
3. *The NeCTAR* (National eResearch Collaboration Tools and Resources) cloud platform,^14^ launched in 2012 by the Australian Research Data Commons, provides Australia’s research community with fast, interactive, self-service access to large-scale computing infrastructure, software and data.
4. *The Mobile-Sandbox* [136] proposed by the University of Erlangen-Nurember, Germany, in 2014 is a static and dynamic analyzer system designed to support analysts detect malicious behaviours of malware.
5. *Credit Card Fraud*. The dataset has been collected and analyzed during a research collaboration of Worldline and the Machine Learning Group (http://mlg.ulb.ac.be) of ULB (Université Libre de Bruxelles) on big data mining and fraud detection [137–145]. The dataset contains transactions made by credit cards in September 2013 by European cardholders. This dataset presents transactions that occurred in two days, where we have 492 frauds out of 284,807 transactions. The dataset is highly unbalanced, the positive class (frauds) accounts for 0.172% of all transactions. It contains only numerical input variables which are the result of a PCA transformation. Unfortunately, the original features and more background information about the data are not provided due to confidentiality issues. The only features not transformed with PCA are ’Time’ and ’Amount’. Feature ’Time’ contains the seconds elapsed between each transaction and the first transaction in the dataset. The feature ’Amount’ is the transaction Amount.
6. *Twitter ISIS Dataset*. The Twitter ISIS dataset [84], published in 2018, consists of ISIS-related tweets/retweets in Arabic gathered from Feb. 2016 to May 2016. The dataset includes tweets and the associated information such as user ID, re-tweet ID, hashtags, number of followers, number of followees, content, date, and time. About 53 M tweets are collected based on the 290 hashtags such as State of the Islamic-Caliphate, and Islamic State. Table 22 provides a brief overview of the Twitter ISIS dataset composition.
7. *Italian Retweets Timeseries*. The Italian Retweets Timeseries dataset [146], published in 2019, contains temporal data of about 5,121,132 retweets from 47,947 users taken from the Italian Twittersphere published between 18/06/2018 and 01/07/2018.

**Table 22:** Twitter ISIS composition

Name	Values
Tweets	9,092,978
Cascades	35,251
Users	1,249,293
Generator users	8056

The breakdown of the usage of the additional datasets in the selected papers is shown in Fig. 6.

### Performance assessment metrics

Frequently, a model’s performance is evaluated by constructing a confusion matrix [147], shown in Table 23, and calculating several metrics from the values of the confusion matrix. Table 24 shows the metrics commonly used to evaluate the performance of ML models. TP represents the true positives, the samples predicted as malicious or attacks that were truly malicious, TN the true negatives, the samples predicted as benign that were truly benign, FP the false positives, the samples predicted as attacks that were in fact benign, and FN the false negatives, the samples predicted as benign that were in fact attacks or malicious.

**Table 23:** Confusion matrix

		Predicted		Total
		Malicious	Benign	
Actual	Malicious	*TN*	*FP*	$TN+FP$
	Benign	*FN*	*TP*	$TP+FN$
Total		$TN+FN$	$TP+FP$	

The accuracy score represents the fraction of correctly predicted samples, benign and malicious, and the error rate considers the misclassified samples. The accuracy metric may be misleading, especially when classes are highly imbalanced. The precision rate is the ratio of correctly predicted benign samples to all samples predicted as benign, and the sensitivity is the ratio of correctly predicted benign samples to samples to all benign samples. The Negative Predictive Value relates to the precision but considers the malicious samples; similarly, the specificity relates to the sensitivity but also considers the malicious samples. The False Positive (Negative) Rate is the ratio of malicious (benign) samples predicted as benign (malicious) to all the malicious (benign) samples. The $F_1$-score is the harmonic mean of the precision and recall scores. This metric aggregates two metrics to provide a more global view of the performance. The Geometric-Mean measures how balanced the prediction performances are on both the majority and minority classes.

**Table 24:** Evaluation metrics

Metric	Equation
Accuracy	$\textbf{ACC}=\frac{TP+TN}{TP+FN+FP+FN}$
Error Rate	$\textbf{ER}=\frac{FP+FN}{TP+FN+FP+FN}$
Precision or positive Predictive value	$\textbf{Precision}=\textbf{PPV}=\frac{TP}{TP+FP}$
Sensitivity or detection rate or true positive rate or recall	$\textbf{TPR}=\textbf{DR}=\frac{TP}{TP+FN}$
Negative predictive value	$\textbf{NPV}=\frac{TN}{TN+FN}$
Specificity or true negative rate	$\textbf{TNR}=\frac{TN}{TN+FP}$
False positive rate or fall out or false acceptance rate	$\textbf{FPR}=\textbf{FAR}=\frac{FP}{FP+TN}$
False negative rate or miss Rate or false rejection rate	$\textbf{FNR}=\textbf{FRR}=\frac{FN}{FN+TP}$
$$F_1$$-score	$$\mathbf {F_1}=\frac{2*Precision*Sensitivity}{Precision+Sensitivity}$$
Geometric-mean	$\mathbf {G-Mean}= \sqrt{Sensitivity*Specificity}$
Cohen Kappa Score	$\mathbf {\kappa }=\frac{p_0-p_c}{1-p_c}$
Matthews correlation coefficient	$\textbf{MCC}=\frac{TP*TN-FP*FN}{\sqrt{(TP+FP)(TP+FN)(TN+FP)(TN+FN)}}$

The kappa ($\kappa$) statistic, introduced in [148], considers a model prequential accuracy, $p_0$, and the probability of randomly guessing a correct prediction, $p_c$. If the model is always correct, $\kappa =1$, and if the predictions are similar to random guessing, then $\kappa =0$. A $\kappa < 0$ indicates less agreement than would be expected by chance alone. The Matthews Correlation Coefficient (also known as phi coefficient or mean square contingency coefficient), introduced in [149], may be seen as a discretization of the Pearson Correlation Coefficient [150], or Pearson’s *r*, for a binary confusion matrix. It measures the difference between predicted and actual values and returns a value between $-1$ and $+1$, where $-1$ indicates a completely incorrect classifier and 1 indicates the exact opposite.

Researchers also use graphical-based metrics to observe the performance. However, these metrics make the comparison between different models more complex. For this reason, summarizations of graphical-based metrics are used. An example of such metrics is the receiver operating characteristic curve, or ROC curve, which provides a graphical representation of a binary classifier system’s diagnostic performance when its discrimination threshold is modified. The Area Under the ROC (AUC ROC or AUROC) represents the probability that a uniformly drawn random positive sample is ranked higher than a consistently drawn random negative sample. Like the ROC, the Precision-Recall Curve (PRC) employs multiple thresholds on the model’s predictions to compute distinct scores for precision and recall. Because computing the Area Under the PRC (AUPRC) is not as straightforward as the AUROC computation process, the interested reader is referred to [151] where a review of the main solutions proposed to compute the AUPRC is presented. Finally, training time and inference time are the time required to build a model and provide predictions, respectively.

**Fig. 7:**
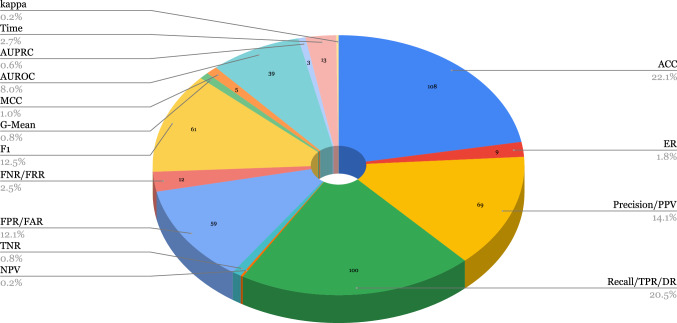
Usage of evaluation metrics in selected papers

As seen in Fig. 7, where we present a breakdown of the usage of the evaluation metrics in the selected papers, the ACC is the most used of the 15 metrics considered for evaluation in the selected papers. In 108 out of the 210 selected papers, or 22.1%, the ACC is used for evaluation. It is followed by the DR which has been used in 100 papers, or 20.5%, the PPV which has been used in 69 papers, or 14.1% and the $F_1$-score which has been used in 61 papers, or 12.5%. As highlighted in Sect. 4.1, except for the “gold standard” dataset, none of the presented datasets is balanced, which points that the ACC measure is not a suitable metric for performance assessment. The DR, PPV and $F_1$-score, however, are more suitable metrics than the accuracy as they consider the class imbalance in datasets. In cyber-security, the DR is useful as there is a high cost associated to attacks, similarly the PPV is an important metric to consider as a low PPV indicates that benign samples or transactions are being flagged as attacks which renders the ML model useless. Due to the imbalanced nature of cyber-security datasets as seen in Sect. 4.1, the $F_1$-score is a useful assessment metric as it simply balances the DR and PPV. The least used metrics are the NPV and $\kappa$-score, which have both been used only once in the selected papers. The NPV is proportional to the frequency of attacks in the dataset, in other words, it is sensitive to imbalanced datasets. As a result, if the prevalence of attacks in the training dataset differs from the prevalence of attacks in the actual world, the computed NPV may be inaccurate. That is, as the prevalence of attacks decreases, the NPV increases because there are more true negatives for every false negative. This is because a false negative would imply that a data point is actually an attack, which is improbable given the scarcity of attacks [152]. Similarly, the $\kappa$-score is also sensitive to imbalanced datasets, therefore it is not suitable in the cyber-security domain where attacks are less frequent than benign samples or transactions. Finally, the time complexity (training and inference) is only reported in 2.7% of the selected papers.

## Open issues and challenges

This section answers our third research question and presents the open challenges found in the literature. We cover open issues and challenges in the areas of the datasets and assessment metrics used, review the learnt lessons and recommend future research directions. Finally, we also discuss the challenge of the gap between research and practice in the field of cyber-security, particularly in the application of ML.

### Datasets and repositories

In Sect. 4.1, we have described 45 datasets, repositories and sources. We summarize the key issues found related to the datasets in this subsection.

***Outdated datasets.***: Over 70 of the 100 reviewed articles focusing on intrusion detection used either the KDD’99 or the NSL-KDD datasets which are closed, anonymized, and outdated (over 20 years old) datasets. Similarly, the most recent Spam and Phishing email dataset used in the selected papers is from 2013. Therefore it is possible that some of the parts under consideration are no longer relevant due to changes in attack vectors and additional factors such as availability and comparability. Additionally, the use of outdated datasets hinders the ability to generalize the results to current real-world scenarios [153].
***Limited number of samples.***: Besides being outdated, both the Spam and Phishing datasets used in the selected papers, except for the TREC and WEBSPAM-UK, contain less data when compared to the intrusion datasets. They comprise 5000 or fewer samples, with the “gold standard” dataset containing only 1600 samples.
***Non-representative data distribution.***: Moreover, in addition to not only containing synthetically generated but also manually labelled data, the class imbalance in these datasets is not representative when compared to real-world scenarios, rendering the proposed approaches ineffective when applied to real data. This is one of the primary reasons why most academic methods are not implemented in practice.
***Lack of train/test splits to allow comparison of results.***: As shown in Table 15, apart from the KDD’99, NSL-KDD, UNIBS2009, AWID2015, UNSW-NB15 and UGR’16 datasets, the datasets in the selected papers are not originally split into train and test partitions, but even then, authors train and test their proposed approaches on random and narrower partitions of these datasets or train/test partitions
***Several datasets used are kept private.***: Most of the data collected from traffic or spam and/or phishing feeds are frequently kept private, making it impossible for other authors to reproduce results.
***Lack of benchmark datasets.***: There are no updated, standard and public benchmark datasets for the different cyber-security problems. Due to these facts, accurate comparisons of the approaches are impossible without having to re-implement them and obtain the data from sources such as traffic or phishing feeds.

In computer science, the quality of the output is decided by the quality of the input, as stated by George Fuechsel in the concept “Garbage in, Garbage out.”. We acknowledge the limitations of the reviewed datasets and repositories and advocate the need for the development of more up-to-date, standardized, and open benchmark cyber-security datasets that reflect the current state of cyber threats and attack vectors, those datasets should also be adequately separated into training/testing and validation partitions. Additionally, we recommend that future studies should consider using multiple datasets and testing the models on a variety of scenarios to improve the generalizability of the results and allow proper evaluation, comparison, and real-world applications.

### Performance assessment metrics

In Sect. 4.2, we presented the 15 metrics used in the selected papers for assessing the performance of the SSL models built on the datasets presented in Sect. 4.1. In this subsection, we present an overview of the significant issues identified in relation to the performance assessment metrics.

***Overlooked/unused metrics.***: Throughout the selected papers, we have noticed that certain important assessment metrics are not used in most of the papers. For example, in [154], only the AUROC is reported, in [155, 156], only DR and FPR or FAR are reported, and in [157] only DR and ACC are reported. This shows that authors are giving more importance to certain metrics while overlooking others, such as PPV and $F_1$-score, which should be used in conjunction as they consider the class imbalance in datasets.
***Inadequate assessment frameworks.***: The accuracy is a misleading metric in imbalanced settings, however, it has been used alone in [158–161]. Furthermore, the accuracy can be inadequate for use in the real world, where data is typically unbalanced. In light of this, it is important to conduct assessments using realistic deployment situations with unbalanced data and adequate assessment frameworks. The chosen metrics must accommodate the needs of the target audience.
***Training and Inference time.***: Only 2.7% reported time complexity measurements, which is an important metric in the cyber-security domain where attack should be detected as soon as possible and static models often need to be rebuilt from scratch to detect unseen attacks, more importance should be given to this assessment metric as it is imperative to detect and mitigate those attacks in a timely manner.
***False alarm rate.***: An excessive amount of false positives may be detrimental to cyber-security because they increase the likelihood that users will ignore or dismiss alarms, leaving them vulnerable to serious cyber threats that they might otherwise have caught. The fact that out of the 210 selected papers, only 59, or 12.1%, measure the FAR–an assessment metric that should be given more weight–demonstrates that it is not being prioritized enough.

The issue of imbalanced data in cyber-security has been the subject of several recent studies. In particular, researchers have explored alternative techniques to address this issue such as cost-sensitive learning [162], which assigns higher costs to the minority class (i.e., the class with fewer instances) than the majority class to encourage the model to focus more on correctly classifying instances of the minority class, thus improving the performance on the rare class. Additional techniques include data augmentation which can be done through methods such as over-/under-sampling, ensemble methods such as bagging and boosting, or using scalar and graphical metrics which are adequate for imbalanced settings [163].

### Bridging the gap between ML-based cyber-security research and practice

The field of cyber-security faces a significant challenge due to the gap between research and practice, especially in the applications of ML [153, 164]. While several industries have successfully deployed ML-based solutions in the field of cyber-security (Sect. 2.3), and research has made significant advances in developing new ML algorithms, the ML algorithms developed by academia are often not practical to implement in real-world scenarios due to scalability, data availability, and regulatory compliance issues. Moreover, the lack of communication and collaboration between academic researchers and industry practitioners adds to the disconnect. As a result, several ML-based cyber-security solutions have not been widely adopted in the industry. This gap underscores the need for increased knowledge sharing and cooperation between researchers and practitioners, a better understanding of the industrial requirements and constraints from academia, as well as a good understanding of ML concepts from both academia and practitioners [165, 166].

To address this gap, there is a need for more interdisciplinary collaboration and partnerships between academia and industry. Collaboration can help researchers better understand the practical challenges faced by practitioners, while practitioners can provide researchers with access to real-world data and feedback on the effectiveness of ML algorithms in practice [164]. Another way to bridge the gap is through the development of standardized evaluation frameworks for ML-based cyber-security solutions as discussed in Sect. 5.2. Standardization can help ensure that ML algorithms are evaluated in a consistent and transparent manner, making it easier for practitioners to understand the effectiveness of a particular solution.

Moreover, it is important to develop ML algorithms that are explainable and interpretable. Several AI algorithms used in cyber-security and other fields, in general, are considered “black boxes” [167], meaning it can be difficult to understand how they make decisions. This lack of transparency can be a barrier to adoption, as it can be difficult for practitioners to trust and validate the results produced by these algorithms. The development of more explainable and interpretable ML algorithms can help address this issue [168–170].

In summary, bridging the gap between research and practice in ML-based cyber-security requires interdisciplinary collaboration, standardized evaluation frameworks, and the development of explainable and interpretable ML algorithms.

## Conclusion

In this survey, we have reviewed the datasets, repositories and performance assessment metrics used in the state-of-the-art applications of SSL methods in the field of cyber-security, namely network intrusion detection, spam and phishing detection, malware detection and categorization, and additional cyber-security areas. Good datasets are necessary for building and evaluating strong SSL models. Our main contribution is an extensive analysis of the cyber-security datasets and repositories. This in-depth analysis attempts to assist readers in identifying datasets and sources that are appropriate for their needs. The review of the datasets reveals that the research community has recognized that there is a lack of publicly available cyber-security datasets and has recently attempted to address this gap by publishing several datasets. Because multiple research organizations are working in this field, further intrusion detection datasets and advancements can be expected in the near future.

We investigated the datasets used in the different papers applying SSL methods for cyber-attack prevention as improvements over conventional security systems and either fully SL or UL methods which would not be adequate in the cyber-security field, where labelled data is often scarce and difficult to obtain. We have reviewed the subcategories of SSL methods and provided a taxonomy based on previous studies. To the best of our knowledge, this is the first work that analyzes the datasets used in the literature applying SSL methods for intrusion, spam, phishing, and malware detection. We have also summarized multiple performance evaluation metrics used for assessing the build models. In addition, where applicable, we have provided brief descriptions, compositions and trends of the datasets used in the reviewed literature. There are no up-to-date and representative benchmark datasets available for each threat domain. However, the datasets reviewed, despite being outdated, are still heavily used in research. Furthermore, most of the publicly available datasets are either imbalanced or not initially split into train/test/validation datasets, making comparing results a tedious task. Moreover, we have outlined the primary open challenges and issues identified in the literature, highlighted strategies for bridging the gap between research and practice, and compiled a comprehensive bibliography in this area. The aforementioned issues and challenges deserve particular attention in future research. Finally, we acknowledge the potential constraints associated with literature reviews, such as limitations on search thoroughness and content selection, which may influence our research; therefore, we made our best efforts to minimize these limitations.

## Footnotes

1 https://securityintelligence.com/new-banking-trojan-icedid-discovered-by-ibm-x-force-research/.

2 https://darktrace.com/news/darktrace-stops-ransomware-attack-at-uk-construction-company.

3 https://feedzai.com/blog/machine-learning-rules-vs-models-in-anti-money-laundering-platforms/.

4 https://bwnews.pr/3YEZbhg.

5 https://www.kaggle.com.

6 https://www.kaggle.com/datasets/shivamb/netflix-shows.

7 https://www.phishtank.com.

8 https://www.phishlabs.com.

9 https://apwg.org.

10 https://www.yelp.com.

11 https://www.delicious.com.au.

12 https://vxug.fakedoma.in/archive/VxHeaven/index.html.

13 http://www.xssed.com/archive.

14 http://www.nectar.org.au/.
